# Health economic evaluation of Autism Adapted Safety Plans: findings on feasibility of tools from a pilot randomised controlled trial

**DOI:** 10.1186/s12913-025-12642-8

**Published:** 2025-03-31

**Authors:** Nawaraj Bhattarai, Jane Goodwin, Mirabel Pelton, Isabel Gordon, Jacqui Rodgers, Sarah Cassidy, Janelle Wagnild, Colin Wilson, Phil Heslop, Emmanuel Ogundimu, Rory C. O’Connor, Sheena E. Ramsay, Ellen Townsend, Luke Vale

**Affiliations:** 1 Health Economics Group, Population Health Science Institute, Newcastle University, Newcastle upon Tyne, NE1 7RU UK; 2 Population Health Sciences Institute, Newcastle University, Newcastle upon Tyne, NE1 7RU UK; 3 School of Psychology, University of Nottingham, Nottingham, NG7 2RG UK; 4 Department of Anthropology, Durham University, Durham, DH1 3LE UK; 5 Social Work, Education and Community Wellbeing, Northumbria University, Newcastle upon Tyne, NE7 7XA UK; 6 Department of Mathematical Sciences, Durham University, Durham, DH1 3LE UK; 7 Suicidal Behaviour Research Lab, School of Health & Wellbeing, University of Glasgow, Glasgow, G12 8TB UK

**Keywords:** Autism, Health economics, Tools, Feasibility

## Abstract

**Background:**

Autism Adapted Safety Plans (AASP) have been proposed to help prevent self-harm and suicidality among autistic adults. The introduction of such plans not only needs to be clinically effective but also cost-effective. The aim of this work was to establish how the cost-effectiveness of AASP could be assessed. Specifically, whether tools and techniques used to collect data for health economic evaluation of the intervention are feasible and acceptable to autistic people.

**Methods:**

A feasibility and external pilot randomised controlled trial of the AASP intervention was conducted. Autistic adults recruited from diverse locations in England and Wales were randomised to either: AASP and usual care, or usual care only. Health economics tools (bespoke and adapted) were developed and focus groups were undertaken with participants, including autistic adults (*n* = 15), their family members/carers (*n* = 5), and service providers (*n* = 10), to determine their acceptability and feasibility. Tools considered worth further exploration were interviewer administered to participants during the pilot trial at baseline and at 6 months. Interviewer notes were used to record any issues reported while completing the tools. Response rates on the questions and completeness of the tools, along with participant feedback in the interviewer notes was assessed.

**Results:**

Standard Gamble and Time-Trade Off approaches to measure health status were judged inappropriate to measure health outcomes with autistic adults experiencing suicidal ideation and with a history of self-harm. Contingent valuation and discrete choice experiments were also considered inappropriate, due to the heavy cognitive burden on respondents. The EQ-5D-5L/VAS, resource utilisation questionnaire and time-travel questionnaire were considered acceptable by participants. Response and completion rates (as a percentage of all returned questionnaires) for resource utilisation questionnaire (> 85%), time-travel questionnaire (> 79%), EQ-5D-5L (> 96%) and EQ-5D-VAS (> 87%) were good in general. Participants needed clear guidance and interviewer support to enable questionnaire completion.

**Conclusions:**

It is feasible and acceptable to collect relevant data on resource utilisation, and costs of accessing care and the EQ-5D-5L in a future definitive trial. Clear guidance and interviewer support on how to complete the questionnaires and explanations of the importance of questions to the research would help autistic participants completing the health economic tools.

**Trial registration:**

ISRCTN70594445; Trial Registration Date: 06/07/2020.

**Supplementary Information:**

The online version contains supplementary material available at 10.1186/s12913-025-12642-8.

## Introduction

Autistic people are more likely to experience poor physical and mental health, alongside self-reported lower quality health care and premature mortality compared with the general population [1–5]. Autistic people have more and, often different, healthcare needs than non-autistic people [2, 6]. However, they can face difficulty in accessing healthcare services [7] and their needs are often unmet [8]. The risk of suicidal thoughts and behaviours is significantly higher among autistic adults compared with non-autistic adults [9–11]. Self-harm is any form of self-injury or self-poisoning irrespective of motive(s), and not necessarily with a suicidal intent [12]. Self-harm, experienced by a high percentage (65%) of autistic adults, is a significant risk marker for suicidal thoughts and behaviours in autistic adults [13]. Correspondingly, death by suicide is higher in autistic people compared with the general population [3, 14]. Therefore, it is important that interventions are identified and tailored to prevent deaths by self-harm and suicide [15] and these interventions are effective in the autistic population. These interventions also need to provide value for money.

Autism Adapted Safety Plans (AASP) are an intervention, specifically adapted in partnership with autistic people and those who support them, to prevent self-harm, suicidal thoughts and suicidal behaviours in autistic adults [16]. Safety plans (SPs) are simple suicide prevention interventions consisting of a prioritised list of hierarchical steps tailored to the individual’s needs to be used in a crisis [16]. SPs have proven efficacy in a range of clinical groups and have the potential for reducing the risk of self-harm and suicide in autistic people [17]. As noted above, there is also a need to determine value for money of AASP. Judgements about value for money can be informed using cost-effectiveness analysis. However, conducting a cost-effectiveness analysis requires additional data on resource utilisation and health outcomes (e.g. QALYs) to be collected and the tools used to collect these data need to be acceptable to autistic people. Data collection instruments created for the general population may not fully capture the intended construct for autistic people, therefore it is recommended that attention should be given to the language and the complexity involved in the tools [18]. Typically, data on health resources utilised by a participant in a defined time period is collected using a resource utilisation questionnaire (www.DIRUM.org), and data on health-related quality of life is collected using EQ-5D tools (e.g., EQ-5D-5L/-VAS [19, 20]) or other tools (e.g. Standard Gamble [19], Time-trade off [19]). In addition, preferences for any outcomes from an intervention are also measured using tools such as contingent valuation method [21] and discrete choice experiments [22].

In any cost-effectiveness analysis consideration needs to be made about which tools are appropriate to answer the research questions posed, which can be achieved by exploring acceptability in feasibility and pilot studies. Such studies are typically underpowered for any form or evaluation and estimates of effectiveness and cost-effectiveness are not a reliable source of evidence for decision making [23, 24]. However they are useful when developing the methods to be used and this can often mean focusing on refining resource utilisation and outcome measure instruments [24]. This includes both the choice of tool, their clarity to respondents, ease of use and completion rates [25]. In addition, consideration needs to be given as to whether they can provide sufficient data to estimate cost-effectiveness. Furthermore, to our knowledge no studies have yet explored whether safety plans could be useful for autistic people and if it is feasible to collect data and conduct economic evaluation of the safety plans intervention. It has been previously acknowledged that there is paucity of economic evaluations on interventions for autism spectrum disorder, partly because of inherent heterogeneity of autistic population and poor availability of valid instruments to collect data for economic evaluation [26, 27]. Therefore, we aimed to establish whether it was feasible to collect data on resource utilisation and outcome measures necessary to conduct an economic evaluation of the AASP intervention, as part of a wider pilot and feasibility study of this intervention [16]. More specifically we determined the acceptability, completeness of resource utilisation and outcome measures that could be used in an economic evaluation conducted as part of a definitive randomised controlled trial (RCT) of AASP with autistic adults.

## Methods

### Tool development

We adapted an existing bespoke (www.DIRUM.org) resource use questionnaire, time and travel questionnaire (to capture the costs of accessing care), a contingent valuation (CV) survey [21], a standard gamble (SG) [19], and a time trade off (TTO) [19] questionnaire for use with autistic adults. Measures of health-related quality of life EQ-5D-5L [20] and EQ-5D-VAS [19, 20] were also considered. We also considered whether a discrete choice experiment (DCE) [22] could be used to capture the preferences of autistic adults for the different outcomes of using the AASP intervention.

### Pretesting tools

We pretested the tools by seeking expert opinion and conducting focus group discussions, to establish their appropriateness (including ease of use, sensitiveness) and feasibility in the study population. The experts providing their opinion were research team members with experience of working with autistic people, clinicians working with autistic people, family members/carers of autistic people and autistic adults themselves.

Full details of the focus group discussions is reported elsewhere [28]. The focus groups consisted of researchers/experts, autistic adults and their family members/carers purposively selected from the information provided by The Autistica Network and third sector mental health and autism organisations in the UK. Participants who consented were invited to take part in discussions organised on a virtual platform Microsoft Teams or Zoom. Between October 2020 and January 2021, six focus group discussions (two with autistic adults, two with family members and two with service providers) were facilitated and conducted by two researchers. The researchers followed a topic guide that explored the acceptability of study design and materials (including potential HE measures). The focus group discussions were recorded and transcribed verbatim and analysed using reflexive thematic analysis as outlined by Braun and Clarke [29]. Thematic analysis refers to a process of exploring and interpreting patterned meaning across qualitative datasets through the “lens” of existing research and theory (whilst still grounded in the data) [30]. Focus group data sets were analysed and coded, with themes derived directly from the data and discussed with the research team to enable consensus where needed.

Tools considered worthy of further exploration following the focus group discussions were pretested to explore their comprehension and ease of completion in a purposive sample of autistic adults (*n* = 8). The feedback from the pre-test was used to refine the questions in these tools.

### Pilot trial

#### Trial summary

A full description of the study is published in the study protocol [16]. The study was a feasibility study and external pilot randomised control trial of a suicide prevention tool aimed at mitigating the risk of self-harm and suicidal behaviour in autistic adults. Autistic adults with experience of self-harm and suicidal thoughts were recruited to the study from multiple diverse locations in England and Wales. Participants in the study were randomised to one of two groups, receiving AASP in addition to usual care or usual care only.

#### Administration of tools

The health economic tools considered worth further exploration on their feasibility were developed into a survey instrument which was administered to participants in the both the arms of the pilot trial at baseline and at 6 months. The tools were administered over video call/telephone (by an interviewer reading out the questions and filling out the participant responses) or sent out via email for the participants to fill out their responses and send back (though only 4 people chose to do it via email at the baseline).

Acceptability was tested by assessing the level of missing data in the data collection forms, the answers filled in by the respondents, and the interviewer notes. The completeness of the data collection forms was assessed using descriptive statistics, with the number and percentage of questionnaires returned along with the nature and extent of missing items within the returned questionnaires. Microsoft Excel was utilised for the descriptive analysis.

#### Interviewer notes

The research interviewer noted any participants comments made while completing the health economics tools and prompted further discussion with open questions if further information/clarity was needed. The interviewer notes were summarised to provide general feedback from the participants on the tools, such as any difficulties and issues in completing them.

## Results

### Appropriateness of tools and refinement

Altogether, 15 autistic adults, 5 family members and 10 service providers took part in the focus group discussions. The majority of focus group participants was female, of white ethnicity, and under 54 years of age. Details of demographic characteristics of the focus group participants are reported elsewhere [28].

Two themes were identified in the thematic analysis. The first theme “Creating the right conditions for developing an AASP” described how autistic adults need to develop an AASP at the right time and place with the right person to support them. The second theme “Creative, flexible and iterative process to develop a personalised AASP” highlights that the process of developing an AASP needs to be flexible (e.g., visual tools, hard copy vs. electronic) and re-visited/adjusted frequently. Details of the thematic analysis is reported elsewhere [28].

Feedback from focus group discussions highlighted the appropriateness of the proposed tools for health outcome measurement and resource utilisation. Considering the sensitive nature of topic (suicidal ideation or self-harm) both the SG and TTO, which typically iteratively use death risks as a trade-off measure in the data generation process, were not considered appropriate tools for measuring health outcomes. The CV and DCE were considered quite complex and were thought to place heavy cognitive burden on respondents regardless of whether they were autistic or not and this burden and the hypothetical nature of the choice process that both use, may accentuate the limitations of these tools [31, 32]. Both tools may also be challenging for those with other aspects of neurodiversity such as dyslexia or dyscalculia and other cognitive difficulties which can be more common amongst those who are autistic compared with the general population [33].

The resource utilisation and time travel questionnaire (see Supplementary files) and EQ-5D-5L (including the EQ-5D VAS) (see Supplementary files) were considered appropriate data collection tools. The feedback from focus group discussions helped to refine and finalise the resource utilisation and time travel questions in terms of asking the right questions on resource utilisation and alternatives to choose from when it came to multiple choice answers. The wording for these tools was found acceptable and clear in general. However, the participants suggested adding some resource utilisation questions on the use of Crisis Resolution and Home Treatment Teams (The Crisis Team), other health care services such as mindfulness, and other services such as an ombudsman and Healthwatch (www.healthwatch.co.uk). The Crisis Resolution and Home Treatment Team is a team of experienced mental health staff, which includes nurses, social workers, psychiatrists and pharmacy staff and offers assessment and home treatment for people over 16 experiencing a mental health crisis, as a short-term alternative to hospital admission. Mindfulness practices are techniques used to improve behavioural and cognitive responses autistic people. Ombudsman is an independent person appointed to look into complaints of services. In the time-travel questionnaire, it was deemed important to add alternatives including “*Just lying down because of ill health*” for the question on what the participant would be doing as their main activity had they not been going to the hospital. It was suggested that “*Just lying down because of ill health*” would reflect a participant’s activity better when they cannot do anything else.

### Pilot trial

#### Participant characteristics

Full details of the participant characteristics are reported elsewhere [34]. A total 49 participants were randomised. Of these 25 were randomised to AASP + usual care and 24 to Usual care trial arms. However, one participant in the AASP + usual care arm was lost to contact after randomisation The mean age of the participants was 39 years and 49% were female. The majority of the participants were White (94%) and had a university or postgraduate education qualification (68%).

#### Feasibility of collecting resource utilisation data

Table 1 presents the summary of the completion of the resource utilisation questionnaire at baseline and follow-up by intervention and comparator groups. The resource utilisation questionnaire covered a range of questions including those related to utilisation of primary care (at GP surgery, home or via telephone/video), crisis resolution and home treatment, emergency services (e.g. NHS111 and A&E visits), hospital admissions and other forms of services (e.g. alternative healthcare services, social services, medication etc.). The completion rates when measured as a proportion of all returned questionnaires were generally high (> 85%) in both arms of the trial. However, the completion rates of questions on medication details were relatively low compared to other questions in the baseline and follow-up; mainly in the responses to the follow-up questionnaire administered at six months (~ 42% in the comparator and ~ 52% in the intervention group at follow-up). The question on medication asked details such as start date, end date, frequency and the dose of the medication. In addition, the question clearly asked the participants only to mention what they could remember. It would generally be hard to recall these medication details and it is reasonable to have lower completion rates for this question. The participants in this study were very committed to providing accurate data and so would rather not answer with a nearest guess and leave it blank when they were not sure. There were no indications that participants with missing responses may not have understood this question on medication.

**Table 1:** Responses for resource utilisation questionnaire at baseline and follow-up

Resource item use	Baseline		Follow-up (6 months)	
	AASP + usual care (*n* = 25)	Usual care (*n* = 24)	AASP + usual care (*n* = 23)	Usual care (*n* = 24)
	Number of complete responses (%)	Number of complete responses (%)	Number of complete responses (%)	Number of complete responses (%)
Primary care/ GP surgery	23 (92%)	22 (91.66%)	20 (86.95%)	21 (87.5%)
Crisis Resolution and Home Treatment	25 (100%)	24 (100%)	21 (91.30%)	23 (95.83%)
NHS111	25 (100%)	23 (95.83%)	21 (91.30%)	24 (100%)
A&E	25 (100%)	24 (100%)	22 (95.65%)	23 (95.83%)
Hospital Admission	25 (100%)	24(100%)	23 (100%)	22 (91.67%)
Alternative treatments (such as Homeopathy, Chinese Medicine, Occupational Health etc.)	25 (100%)	23 (93.85%)	20 (86.95%)	24 (100%)
Home Assistance- Social Services	24 (96%)	23 (95.83%)	21 (91.30%)	24 (100%)
Other services (e.g. Ombudsman, Legal, Police, Healthwatch etc.)	24 (96%)	23 (95.83%)	23 (100%)	23 (95.83%)
Medication	18 (72%)	17 (73.91%)	12 (52.17%)	10 (41.67%)

#### Feasibility of collecting time-travel data

Table 2 presents the summary of completion rates of the time and travel questionnaire at baseline and follow-up. The time and travel questionnaire asked participants how long it took them to travel to access their recent primary care/hospital service, how they travelled, and what they would be doing if they were not visiting the healthcare service. The completion rates as a proportion of all returned questionnaires were > 79% in both comparator and intervention groups at baseline and follow-up. We did not see any evidence to suggest that the participants did not understand the questions on time and travel.

**Table 2:** Responses for time and travel questionnaire-baseline and follow-up

Resource item use	Baseline		Follow-up (6 months)	
	AASP + usual care (*n* = 25)	Usual care (*n* = 24)	AASP + usual care (*n* = 23)	Usual care (*n* = 24)
	Number of complete responses (%)	Number of complete responses (%)	Number of complete responses (%)	Number of complete responses (%)
GP practice	23 (92%)	22 (91.66%)	20 (86.95%)	24 (100%)
Hospital	22 (88%)	19 (79.16%)	20 (86.95%)	21 (87.5%)

#### Feasibility of collecting EQ-5D data

Tables 3 and 4 present the summary of the responses on EQ-5D-5L at baseline and at follow-up respectively. Examining the completion rates of participants, it was evident that all participants were able to complete the EQ-5D-5L questionnaire at baseline (*n* = 25 for intervention; *n* = 24 for comparator) and follow-up (*n* = 23 for intervention; *n* = 24 for comparator), except one participant in the intervention group who at baseline did not provide responses in the usual activities domain. However, the EQ-5D VAS was not completed by 4% and 12.5% in the intervention group and comparator groups respectively at baseline. At follow-up, all participants in the comparator group completed the EQ-5D VAS, however 4.3% in the intervention group did not. These completion rates may suggest that the questionnaire may need improved guidance on how the VAS scale should be scored on a scale of 0–100. Difficulties in completing the EQ-5D-VAS have been discussed further below in the section “Interviewer notes”.

The percentage of participants responding to each dimension and levels of EQ-5D-5L are presented in Tables 3 and 4. The percentage of participants reporting some problems (any problem) across domains largely remained similar to baseline at follow-up in both the groups. Similarly, the changes in percentage of individuals reporting each level (ranging from no problems to unable/extreme) for each domain at both baseline and follow-up did not appear to differ. There were, however, higher increases (from baseline to follow up) in the percentage of participants reporting moderate problems in self-care and usual activities in the control compared with the intervention group. At follow-up (Table 4), the percentage of participants reporting moderate problems in the pain/discomfort and anxiety/depression domains decreased from baseline (Table 3) in the control group whereas these increased in the intervention group. Nevertheless, care should be taken not to over-interpret these data given the small sample size of the pilot study.

**Table 3:** Responses for EQ-5D-5L-baseline

Level*	Mobility, *n* (%)		Self-care, *n* (%)		Usual activities, *n* (%)		Pain/Discomfort, *n* (%)		Anxiety/depression, *n* (%)	
	AASP + usual care	Usual care	AASP + usual care	Usual care	AASP + usual care	Usual care	AASP + usual care	Usual care	AASP + usual care	Usual care
1	15 (60%)	11 (45.83%)	15 (60%)	10 (41.67%)	11 (45.83%)	12 (50%)	7 (28%)	5 (20.83%)	2 (8%)	7 (30.43%)
2	4 (16%)	5 (20.83%)	2 (8%)	10 (41.67%)	4 (16.67%)	5 (20.83%)	8 (32%)	7 (29.17%)	7 (28%)	5 (21.74%)
3	3 (12%)	6 (25%)	4 (16%)	3 (12.50%)	4 (16.67%)	5 (20.83%)	5 (20%)	9 (37.5%)	7 (28%)	8 (34.78%)
4	3 (12%)	1 (4.17%)	4 (16%)	1 (4.17%)	5 (20.83%)	1 (4.17%)	5 (20%)	1 (4.17%)	7 (28%)	2 (8.69%)
5	0 (0%)	1 (4.17%)	0 (0%)	0 (0%)	0 (0%)	1 (4.17%)	0 (0%)	2 (8.33%)	2 (8%)	1 (4.35%)
Total (n)**	25 (100%)	24 (100%)	25 (100%)	24 (100%)	24 (100%)	24 (100%)	25 (100%)	24 (100%)	25 (100%)	23 (100%)
Participants reporting some problems (%)***	10 (40%)	13 (54.17%)	10 (40%)	14 (58.33%)	13 (54.17%)	12 (50%)	18 (72%)	19 (79.17%)	23 (92%)	16 (69.57%)

*1-no problems; 2- slight problems; 3- moderate problems; 4- severe problems; 5– unable/extreme

**VAS score: Not complete in AASP + usual care = 1 (1/25 = 4%); Usual care = 3 (3/24 = 12.5%)

***Any problem

**Table 4:** Responses for EQ-5D-5L-follow-up

Level*	Mobility, *n* (%)		Self-care, *n* (%)		Usual activities, *n* (%)		Pain/Discomfort, *n* (%)		Anxiety/depression, *n* (%)	
	AASP + usual care	Usual care	AASP + usual care	Usual care	AASP + usual care	Usual care	AASP + usual care	Usual care	AASP + usual care	Usual care
1	14 (60.87%)	12 (50%)	12 (52.17%)	11 (45.83%)	7 (30.43%)	8 (33.33%)	7 (30.43%)	6 (25%)	2 (8.70%)	6 (25%)
2	3 (13.04%)	2 (8.33%)	1 (4.35%)	7 (29.17%)	6 (26.09%)	3 (12.5%)	5 (21.74%)	10 (41.67%)	2 (8.70%)	8 (33.33%)
3	5 (21.74%)	7 (29.17%)	8 (34.78%)	5 (20.83%)	5 (21.74%)	9 (37.5%)	5 (21.74%)	4 (16.67%)	8 (34.78%)	4 (16.67%)
4	1 (4.35%)	2 (8.33%)	1 (4.35%)	1 (4.17%)	4 (17.40%)	3 (12.5%)	4 (17.40%)	4 (16.67%)	10 (43.48%)	6 (25%)
5	0 (0%)	1 (4.17%)	1 (4.35%)	0 (0%)	1 (4.35%)	1 (4.17%)	2 (8.70%)	0 (0%)	1 (4.35%)	0 (0%)
Total (n)**	23 (100%)	24 (100%)	23 (100%)	24 (100%)	23 (100%)	24 (100%)	23 (100%)	24 (100%)	23 (100%)	24 (100%)
Participants reporting some problems (%)***	9 (39.13%)	12 (50%)	11 (47.83%)	13 (54.17%)	16 (69.57%)	16 (66.67%)	16 (69.57%)	18 (75%)	21 (91.30%)	18 (75%)

*1-no problems; 2- slight problems; 3- moderate problems; 4- severe problems; 5– unable/extreme

**VAS score: Not complete in AASP + usual care = 1 (1/23 = 4.34%); Usual care = 0 (0/24 = 0%)

***Any problem

#### Interviewer notes

The interviewer notes from the pilot trial allowed us to understand what participants thought of about completing the health economics tools. Whilst a high percentage of participants were able to complete the health economics tools, some participants told us that these questionnaires felt like an interrogation and quite demanding (participant quote “*question felt like too much*”) in terms of the extent of information asked. It was challenging for the participants to remember the details of the services accessed in the last six months, travel time taken to access any service or the medication dosage (their start and end dates) they take. Participants felt that the information needed to be accurate despite being made aware by the researcher (interviewer) that it could be any nearest estimate they could remember. As mentioned earlier, the participants were committed to providing accurate responses and would rather not answer than provide misleading data. Participants commented that they felt that the study was important, however some participants felt remembering and estimating answer details was very anxiety provoking. The researcher had to assist participants to put in their best estimate and reassure them it was okay and understandable if they were unsure of answers to some questions (for example, how many times they saw their GP) and did not want to put a guess answer (to avoid any anxiety later if they realise the answer was not accurate). Participants also questioned why the details on the resource utilisation questions were important, and why these details could not be taken from their patient records instead of asking them directly. Some participants, felt like there was limited information on the questions and instructions on the expected answer for the questions, and how much time it needed to complete these questionnaires. There were instances where participants did not understand what a particular healthcare/social service meant (for example, crisis resolution and home treatment team, mindfulness), possibly because those services were not available in their area or had different titles. In the time-travel questionnaire, one participant answered as “*no fee*” to the question on cost of car use to the GP appointment suggesting that cost involved in general (e.g., for fuel or anything involved with the car use) would be more appropriate to ask. Some participants reported that trying to interact with health services costs time and energy, however these were not recorded in the questionnaire. For example, spending an hour in call waiting when contacting health services, co-ordinating with a support person to make calls for them, or being unable to see the GP for health concerns due to anxiety about speaking on the telephone which could cost them work time due to extended sick leave. Participants felt that it is important to record these costs of trying to arrange/contact health services.

Participants also were not clear on the EQ-5D-5L dimensions and needed some instructions explaining what these dimensions were (and they felt it needed clarity as to whether it is autism specific or more general). Participants also found it hard to compare themselves to people who may have terminal illness and/or people with very good physical and mental health because they didn’t know what that felt like. Finally, participants found the EQ-5D-5L question on anxiety/depression hard to separate and thus respond to (for example, low depression, high anxiety - how do they respond when the question combines them? ). There were issues around completing the EQ-5D-VAS, as some participants found it hard to understand what health meant and found it difficult to score between 0 and 100 when their physical health and mental health were very different (for example, physically very well, mentally extremely anxious). Putting a score to their health on the day of interview was hard for some participants because they may have been having a particularly good or bad day that was not representative of their experience and they felt it would skew the data. Research interviewers spent a lot of time discussing how to arrive at an answer and explaining what the questions mean, therefore some participants found easier to complete questionnaires with the additional interviewer support and guidance.

Overall, our findings showed that it would be feasible and appropriate to collect data from autistic participants using the resource utilisation, time-travel questionnaire and EQ-5D-5L (including the EQ-5D-VAS) in a future definitive trial.

## Discussion

There is a need to assess the cost-effectiveness and to determine the value for money of interventions designed to prevent suicide and self-harm in autistic people. It is important to ascertain which tools are appropriate and acceptable to research participants, and whether it is feasible to collect sufficient data to conduct a cost-effectiveness analysis.

We evaluated the feasibility of collecting data on outcomes and resource utilisation with autistic adults and their carers in this study. Completion rates for each item in the EQ-5D-5L, EQ-5D-VAS and a bespoke resource-use and time travel questionnaire were evaluated at baseline and follow-up (6 months). The study also sought expert opinion on the appropriateness of SG, TTO, CV, and DCE as other forms of outcome measures along with conducting focus group discussions of representative autistic adults, experts and researchers.

Considering the sensitive nature of suicidal ideation and self-harm, SG and TTO were judged inappropriate to use as a health outcome measure as they ask about what risk of death or time spent in full health as a way of valuing states of health less than full health. CV and DCE are quite complex and are generally considered to have a heavy cognitive burden on the participants. In other studies, approaches such as a DCE have been used with some success. For example, in the Transitions Project [35] a cohort of young adults with autism (the term they preferred to use) completed a DCE looking at preferences for how services might be organised as they moved from child to adult services. In this study the DCE was completed as part of an interview, and it was supported by a set of aids to help respondents think about their responses to the DCE questions. Such an approach may not be practical in a large-scale RCT.

Autistic participants in both arms of the trial were more likely to respond to resource utilisation, time travel and EQ-5D questionnaires. We acknowledge that the high response rate on theses questionnaires could be because they were interviewer administered over the telephone or via email (because of COVID-19 restrictions in place then). The completion rates for these questionnaires were good overall, supporting that these questions were clear in terms of wording and were easy to complete. We acknowledge that 98% of participants in our pilot trial were “White” and 68% of the participants had a college degree/postgraduate qualifications, where education attainment and ethnicity might have influenced the high completion rates observed in our study [36]. However, a few participants in both the arms of the trial could not complete the EQ-5D-VAS. This finding is not uncommon, as previously the EQ-5D-VAS in the UK NHS patient reported outcome measures (PROMs) programme has been found challenging to complete and less than 50% of respondents completed as per the instructions [37].

Perhaps the responses on the EQ-5D-VAS could be improved with better guidance on how to interpret the question and complete the scoring [37]. With respect to resource use, incomplete responses on some of the items such as medication use could be because of participants finding it hard to recall the details on the dosing/start and end date of a particular medication. These incomplete responses are equally present in both arms of the trial making the risk of bias minimal. A simplified form of data collection for medications might work better if standard dosages and durations could be defined beforehand with practitioners and experts by experience.

The interviewer notes clearly indicate that the participants felt that the health economics tools asked too much detail, lacked clarity in places and were time consuming. It could be possible to identify ways to shorten the length of the questionnaires (for example getting information from patient records where possible). However, previous studies have shown that the clarity of questionnaires is likely to be associated with response burden rather than questionnaire length [38]. Therefore, it is important that participants are informed clearly beforehand how long it may take them to complete the questionnaires, what the questions would involve and why it is important for the researchers to ask those questions. Providing more guidance (in text and by the interviewer) on the questions may be helpful. Providing reassurance that it is fine if they could not remember all details (for example, the healthcare services accessed in the last 6 months, time taken to them, or the medication dosage including their start date and end date) and it is normal to have to look up this information. However, it is important not to cause unnecessary participant distress if this is too difficult or time consuming for them. Participants need reassurance that their wellbeing is more important than getting the information right and they should not be experiencing a lot of anxiety about calling the GP to check for details or accurate estimates. It also important to account for the costs of time spent while trying to interact/contact with health services and resulting extended ill-health associated with anxiety of speaking with health services in travel-time questionnaire in a definitive trial. EQ-5D-5L/-VAS are copyrighted instruments and their wording and style cannot be modified however, autistic participants might find it useful to have more specific guidance when completing the EQ-5D-5L (for example, highlighting that the health dimensions are not autism specific) and EQ-5D-VAS (for example, showing them how it is scored and stating that the score they provide will suggest how good their general health is on that day). In general, participants maybe more likely to feel comfortable filling out these questionnaires if they understand the importance of the questions (and details) for the future definitive trial.

The key finding from this study is that it would be feasible to collect data from autistic participants using the resource utilisation, time-travel questionnaire and EQ-5D-5L in the future definitive trial. However, the percentage changes (at follow-up from baseline) to participants responding to EQ-5D-5L in each of study groups may not be sufficient to show that EQ-5D-5L would be a responsive generic health outcome measure for autistic adults. EQ-5D has been reported to show low to medium responsiveness previously [39, 40] and thus there could be a chance that it may not represent changes in health status of some participant groups with certain conditions [41]. An alternative to EQ-5D-5L could be capability based instruments such as ICECAP recommended by NICE [42]. However, caution should be exercised as this study was not adequately powered to detect such responsiveness, and full RCT may be needed for EQ-5D-5L to detect changes in autistic adults’ health over time. It should also be noted that both the ICECAP and EQ-5D-5L ask about health of the respondents on that specific day and may not be practical to ask respondents to complete these tools frequently enough to capture changes in health conditions which change rapidly over time. A lot of time was spent by research interviewers discussing how to arrive at an answer and what the questions meant (for example, what the question is asking versus potential interpretations of the phrasing), therefore completing the questionnaire with a research interviewer may be easier for some autistic adults than filling it out by themselves. A generally high response rate to the questions and completeness of the tools could be because of the interviewer support to the participants completing the tools. It is important that researcher time to support participants to complete the questionnaire (though there should be an option to complete it themselves) should be factored into the cost of future definitive trial. Finally, our study sample was predominantly white and with post-graduate qualification; therefore, not necessarily representative of the autism population generally, therefore, our generalisability of our findings should be interpreted with caution. Economic evaluations on interventions for autism are sparse and methodologically limited [26]. Whilst our findings would be important in conducting economic evaluations on interventions for autism spectrum disorder, mainly in a definitive trial on AASP, we acknowledge that there could be challenges in terms of heterogeneity of autistic population and appropriateness of the instruments and outcomes [27]. Therefore, we recommend that future economic evaluations consider our findings in the context of the differences in the sample population characteristics, the ease and acceptability of instruments to collect health economic data, and the appropriateness of the outcomes.

## Conclusions

The results of the feasibility assessment and piloting of health economic evaluation tools could provide the information for an economic evaluation conducted as part of a prospective full-scale RCT. SG and TTO would not be appropriate tools to measure generic health outcomes in autistic adults who already have suicidal ideation and instances of self-harm. CV and DCE were not felt to be appropriate, due to their heavy cognitive burden. Completion rates for resource utilisation and time travel questionnaire, and EQ-5D-5L/EQ-5D-VAS are good overall indicating that it would be feasible to collect resource use and health outcomes data using these tools in a definitive RCT. However, the current study does not provide sufficient evidence to show if EQ-5D-5L would be responsive in an autistic adult sample. Clear guidance on the questionnaire and explaining the importance of questions to the research could be helpful for the participants completing the questionnaire.

## Supplementary Information

Supplementary Material 1.

## Data Availability

No datasets were generated or analysed during the current study.
