# Supplementary material for: Health economic evaluation of Autism Adapted Safety Plans: findings on feasibility of tools from a pilot randomised controlled trial
Source: BMC Health Serv Res. 2025 Mar 31;25:473. doi: 10.1186/s12913-025-12642-8 (PMC11956318; doi:10.1186/s12913-025-12642-8)
Supplement: Supplementary file 1 — Supplementary Material 1. [file 12913_2025_12642_MOESM1_ESM.docx]

Participant ID

|  |  |  |  |  |  |  |  |  |  |  |  |  |  |  |  |  |  |  |  |
| --- | --- | --- | --- | --- | --- | --- | --- | --- | --- | --- | --- | --- | --- | --- | --- | --- | --- | --- | --- |
|  |  | **Date you filled in this questionnaire** | | | | | | **D** | **D** | **/** | **M** | **M** | **/** | | **Y** | **Y** | | **Y** | **Y** |


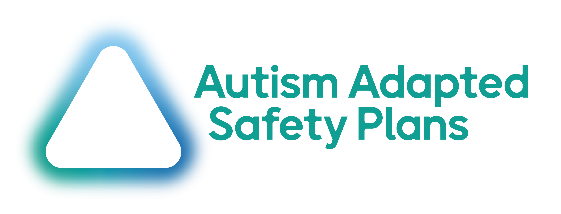


**RESOURCE UTILISATION FORM**

**V1**

**3/6/21**

**Thank you for helping us with our research.**

**We would be very grateful if you could complete this questionnaire.**

**CONFIDENTIAL**

Baseline/6 months

**Instructions:** We need to ask you some questions relating to your use of health care services and the medications that you currently take. We are doing this to find out about the costs of different treatments to the National Health Service, Social Care and other sectors.

The following questionnaire is broken down into two sections (Section A and Section B, as detailed below). Some questions will seem more relevant to you than others, but please try to answer all the questions.

Some of questions ask you to indicate your answers by placing a tick **(✓)** in the appropriate box, and other questions ask you to write your response in the box provided. If you are unsure about any answer, please write in your best guess. Please also feel free to note any additional comments beside the relevant questions.

The sections covered in this questionnaire are as follows:

**Section A: Your Use of Health Care and Social Services**

**Section B: Your Medication**

The information you provide will be **completely confidential**. Your answers will be combined with the answers of other participants involved in the study and reported in such a way that it will not identify you.

**Section A – Your Use of Health Care and Social Services**

This section is about **your use of health care services in the last 6 months**. We know that it can be difficult to remember the services you have used precisely, but please be as accurate as you can. In order to avoid duplication, it is important that you tell us about your use of healthcare services only once. If you are unsure about how to complete the questionnaire, please contact the research team.

**A1. In the last 6 months**, have you accessed any health care services for yourself?

Yes No

**If you have** accessed health services in the past 6 months, in the boxes below can you please indicate how many times in the past 6 months you have consulted the following professionals? **The number of times could be approximate if you cannot remember exactly.**

**If you have not** accessed any health services in the past 6 months, please move on to question A2 (page 4).

|  | **Seen** | **At Home** | **At GP Practice/Hospital/Other Venues** | **By Phone/Video Call** |
| --- | --- | --- | --- | --- |
| General Practitioner (GP) | Yes/No |  |  |  |
| Community Nurse | Yes/No |  |  |  |
| Occupational Therapist | Yes/No |  |  |  |
| Physiotherapist | Yes/No |  |  |  |
| Speech & Language Therapist | Yes/No |  |  |  |
| Dietitian | Yes/No |  |  |  |
| NHS Psychologist | Yes/No |  |  |  |
| Improving Access to Psychological Therapies (IAPT) services such as Cognitive Behavioural Therapy (CBT), Talking therapies | Yes/No |  |  |  |
| NHS Counsellor | Yes/No |  |  |  |
| Social Worker | Yes/No |  |  |  |
| Community Psychiatrist | Yes/No |  |  |  |
| Community Psychiatric Nurse | Yes/No |  |  |  |
| Drug and alcohol services | Yes/No |  |  |  |
| Pharmacist | Yes/No |  |  |  |
| Phlebotomist | Yes/No |  |  |  |
| Other (s): (please write in the space provided) |  |  |  |  |
|  |  |  |  |  |
|  |  |  |  |  |
|  |  |  |  |  |
|  |  |  |  |  |
|  |  |  |  |  |

**A2. In the last 6 months** have you contacted the Crisis Resolution and Home Treatment Team (The Crisis Team) for any problems you faced?

| Yes No |  |
| --- | --- |
| **If you have** contacted The Crisis Team in the last 6 months please record the number of times here:  **In this and the following questions, the number of times could be approximate if you cannot remember exactly.** | |
|  | |

**A3. In the last 6 months** have you contacted NHS 111 for any health problems you faced?

| Yes No |  |
| --- | --- |
| **If you have** contacted NHS 111 in the last 6 months please record the number of times here: | |

**A4. In the last 6 months** have you attended accident and emergency (A&E)?

| Yes No |  |
| --- | --- |
| **If you have** attended accident and emergency (A&E) in the last 6 months please record the number of times here: | |

**A5. In the last 6 months** have you been admitted to hospital?

Yes No

**If you have** been admitted to hospital in the last 6 months, please record details onto the following table. If you have stayed overnight, please tell us how many nights you stayed in hospital overall. Please consider all hospital admissions, even if those were in multiple hospitals.

**If you have not** been admitted to hospital in the last 6 months, please move on to question A6 (page 6).

| **Hospital visits** | | |
| --- | --- | --- |
| 1. Have you used an emergency ambulance service to attend the hospital? | Yes No | If yes, number of times |
| 1. Have you been admitted to hospital as a day patient? | Yes No | If yes, number of times |
| 1. Have you had an **emergency** admission to hospital as an inpatient (stayed in hospital overnight or longer)? | Yes No | If yes, number of **nights**  you stayed in hospital  overall |
| 1. Have you had a **planned/arranged** admission to hospital as an inpatient (stayed in hospital overnight or longer)? | Yes No | If yes, number of **nights**  you stayed in hospital  overall |

**A6. In the last 6 months**, have you used any other health care services or alternative services?

Yes No

**If you have** used any other health care services in the last 6 months, in the boxes below can you please indicate how many times in the past 6 months you have consulted the following services? **The number of times could be approximate if you cannot remember exactly**.

**If you have not** used any other health care services in the last 6 months, please move on to question A7 (page 7).

|  | **Seen** | **At Home** | **Practice/Hospital** | **By Phone** |
| --- | --- | --- | --- | --- |
| Homeopathy | Yes/No |  |  |  |
| Traditional Chinese Medicine | Yes/No |  |  |  |
| Private Counselling | Yes/No |  |  |  |
| Private Occupation Therapy | Yes/No |  |  |  |
| Private Speech and Language | Yes/No |  |  |  |
| Mindfulness | Yes/No |  |  |  |
| Aromatherapy | Yes/No |  |  |  |
| Other(s): (Please write in the space provided) |  |  |  |  |
|  |  |  |  |  |
|  |  |  |  |  |
|  |  |  |  |  |
|  |  |  |  |  |
|  |  |  |  |  |
|  |  |  |  |  |

**A7. In the last 6 months**, have you received any home assistance from social services?

Yes No

**If you have** received any home assistance in the last 6 months, please record details onto the following table. **The number of times could be approximate if you cannot remember exactly**.

**If you have not** had any home assistance in the last 6 months, please move on to question A8.

| **Social Services** | | | | |
| --- | --- | --- | --- | --- |
| **Type of service** | **No** | **Yes** | **Frequency**  **(If Yes)** | **Total in last 6 months** |
| 1. Home help visited to help me with personal care |  |  | Number of home visits |  |
| 1. Home help visited to help me with household tasks |  |  | Number of home visits |  |
| 1. Home help did shopping for me |  |  | Number of times |  |
| 1. Other services, please state: |  |  | Number of times |  |

**A8. In the last 6 months**, have you used **any other services** such as the services below?

Yes No

**If you have** used any other services in the last 6 months, please record details onto the following table. **The number of times could be approximate if you cannot remember exactly.**

**If you have not** used any other services in the last 6 months, please move on to question B1 (page 9).

|  | **Contacted** | **At Home** | **Their Office Building/Other locations** | **By Phone/Video call** |
| --- | --- | --- | --- | --- |
| Ombudsman | Yes/No |  |  |  |
| Advocacy | Yes/No |  |  |  |
| Legal | Yes/No |  |  |  |
| Police | Yes/No |  |  |  |
| Healthwatch | Yes/No |  |  |  |
| Other(s): (Please write in the space provided) |  |  |  |  |
|  |  |  |  |  |
|  |  |  |  |  |
|  |  |  |  |  |
|  |  |  |  |  |
|  |  |  |  |  |
|  |  |  |  |  |
|  |  |  |  |  |
|  |  |  |  |  |
|  |  |  |  |  |

**SECTION B – Your Medication**

**B1. In the last 6 months** have you taken any medications prescribed by a health care professional?

**If you have** taken any medications prescribed by a health care professional in the last 6 months, please record details onto the following table. **Please write what you can remember.**

**If you have not taken** any medications, please move on to question B2 (page 11).

| **Drug name** | **Drug dosage** | **Frequency** | **Start date** | **Stop date**  **(if applicable)** |
| --- | --- | --- | --- | --- |
| *Example: Aspirin* | *75mg* | *Once per day* | *1/4/2019* |  |
| **1)** |  |  |  |  |
| **2)** |  |  |  |  |
| **3)** |  |  |  |  |
| **4)** |  |  |  |  |
| **5)** |  |  |  |  |
| **6)** |  |  |  |  |
| **7)** |  |  |  |  |
| **8)** |  |  |  |  |
| **9)** |  |  |  |  |
| **10)** |  |  |  |  |

**B2.** Did anyone help you fill in this questionnaire today?

Yes No

**THANK YOU**

**This is the end of the questionnaire. Please could you take a moment to check that you have answered as many questions as possible.**

**We would like to thank you for your time spent completing the questionnaire. The information you have given us will be extremely useful in helping us carry out our research.**

**It will be treated with the strictest confidence and kept securely.**

Participant ID

|  |  |  |  |  |  |  |  |  |  |  |  |  |  |  |  |  |  |  |  |
| --- | --- | --- | --- | --- | --- | --- | --- | --- | --- | --- | --- | --- | --- | --- | --- | --- | --- | --- | --- |
|  |  | **Date you filled in this questionnaire** | | | | | | **D** | **D** | **/** | **M** | **M** | **/** | | **Y** | **Y** | | **Y** | **Y** |


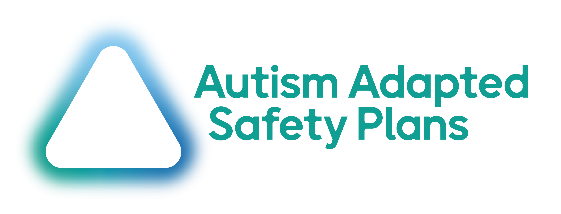


**TIME AND TRAVEL QUESTIONAIRE**

**V1**

**3/6/21**

**Thank you for helping us with our research.**

**We would be very grateful if you could complete this questionnaire.**

**CONFIDENTIAL**

Baseline/6 Months

# **THE QUESTIONNAIRE: YOUR TIME AND TRAVEL SPENT ON HEALTHCARE APPOINTMENTS**

**Instructions:** The following questionnaire is broken down into two sections (Section A and Section B, as detailed below). Some questions will seem more relevant to you than others, but please try to answer all the questions.

The information will help us work out the costs to you and your family of using health care. Some of questions ask you to indicate your answers by placing a tick **(✓)** in the appropriate box, and other questions ask you to write your response in the box provided.

Please read the questions carefully and answer each one as accurately as you can. **If you are unsure about any answer, please write in your best guess**. Please also feel free to note any additional comments beside the relevant questions.

The sections covered in this questionnaire are as follows:

**Section A: Visiting the GP Practice**

**Section B: Visiting the Hospital**

The information you provide will be **completely confidential**. Your answers will be combined with the answers of other patients involved in the study and reported in such a way that it will not identify you or influence your pattern of treatment.

***Section A – Visiting the GP Practice***

This section is about your **most recent** visit to your *GP practice* to see the GP or the practice Nurse only for yourself.

**A1. Have you visited the *GP practice* for any reason in the last 6 months?**

Yes No

If **you have** visited the GP practice in the last 6 months please complete the remainder of Section A below.

***We would like you to think of your last visit to your GP practice for yourself.***

If you **have not** visited your local GP practice (e.g. to see the GP and/or the practice nurse etc) in the last 6 months, please go to Section B (page 5).

**A2. Approximately how long did you spend at the *GP practice*?**

This is inclusive of time spent waiting to be seen, time spent with health care professionals and any time to arrange other appointments.

| Hours | \|  \|  \| \| --- \| --- \| | Minutes | \|  \|  \| \| --- \| --- \| |
| --- | --- | --- | --- | --- | --- | --- | --- |

**A3. How did you travel to the *GP practice*?**

If you used more than one form of transport, **please indicate the way you travelled for the main** (longest in terms of distance) part of your journey.

If you travelled by public transport (e.g. bus or train) or by taxi for part or the entire journey, **please write the cost of the return fare** in the ‘Cost’ column in pounds and pence.

If you took a car, **please write the usual parking fees per visit** in the ‘Cost’ column in pounds and pence. **Please give only parking fees and not the other costs of using a car.**

If you cannot remember exactly, please indicate an approximate cost.

| **Method of transport** | **Tick one category** | **Cost** |
| --- | --- | --- |
| Walk | 🞏 |  |
| Car | 🞏 | £___.___ |
| Public Transport | 🞏 | £___.___ |
| Taxi | 🞏 | £___.___ |
| Ambulance | 🞏 |  |
| Other, please state _____________________ |  | £___.___ |

**A4. Approximately how long did your journey to the *GP practice* take *(one way)*?**

| Hours | \|  \|  \| \| --- \| --- \| | Minutes | \|  \|  \| \| --- \| --- \| |
| --- | --- | --- | --- | --- | --- | --- | --- |

**A5. Approximately how far did you have to travel to visit the *GP practice (one way)*?**

Please give the total distance in miles for the journey to the GP practice (one way). Please write approximate distance even if it is less than a mile (e.g. 0.5 or 0.2 mile).

| Journey distance in miles | \|  \|  \|  \| \| --- \| --- \| --- \| |
| --- | --- | --- | --- | --- |

**A6. What would you have been doing as your main activity if you had not been going to the *GP practice*?**

| Paid work |  | Caring for someone |  |
| --- | --- | --- | --- |
| Childcare |  | Leisure activities |  |
| Housework |  | Voluntary work |  |
|  |  | Study |  |
|  |  | Just lying down because of ill health |  |

Other- please give details:

**A7. Did you travel to the *GP practice* on your own or were you accompanied by another person?**

Please tick one box.

| By myself |  |
| --- | --- |
| Accompanied by a support person (e.g., relative/friend/carer) |  |

***Section B – Visiting the Hospital***

**B1. Have you been to a hospital for a healthcare appointment (for any reason) in the last 6 months?**

***We would like you to think of your most recent visit in the last 6 months to a hospital for yourself including any A&E visits.***

Yes No

**If you have** visited a hospital in the last 6 months, please complete the remainder of Section B below.

**If you have not** visited a hospital in the last 6 months, please move on to B8 (page 7).

***We would now like you to think about your last appointment at the hospital***

**B2. Approximately how long did you spend at the hospital?**

This is inclusive of time spent waiting to be seen, time spent with health care professionals and any time to arrange other appointments.

| Hours | \|  \|  \| \| --- \| --- \| | Minutes | \|  \|  \| \| --- \| --- \| |
| --- | --- | --- | --- | --- | --- | --- | --- |

**B3. How did you travel to the hospital?**

If you used more than one form of transport, please indicate the way you travelled for the main (longest in terms of distance) part of your journey.

If you travelled by public transport (e.g. bus or train) or by taxi for part or the entire journey, please write the cost of the return fare in the ‘Cost’ column in pounds and pence. If you took a car, please write the usual parking fees per visit in the ‘Cost’ column in pounds and pence. Please give only parking fees and not the other costs of using a car.

If you cannot remember exactly, please indicate an approximate cost.

| **Method of transport** | **Tick one category** | **Cost** |
| --- | --- | --- |
| Walk | 🞏 |  |
| Car | 🞏 | £___.___ |
| Public Transport | 🞏 | £___.___ |
| Taxi | 🞏 | £___.___ |
| Ambulance | 🞏 |  |
| Other, please state _____________________ |  | £___.___ |

**B4. Approximately how long did you your journey take to the *hospital* take *(one way)*?**

| Hours | \|  \|  \| \| --- \| --- \| | Minutes | \|  \|  \| \| --- \| --- \| |
| --- | --- | --- | --- | --- | --- | --- | --- |

**B5. Approximately how far did you have to travel to visit the hospital (one way)?**

Please give the total distance in miles for the journey to the hospital (one way).

| Journey distance in miles | \|  \|  \|  \| \| --- \| --- \| --- \| |
| --- | --- | --- | --- | --- |

**B6. What would you have been doing as your main activity if you had not been going to the hospital?**

| Paid work |  | Caring for someone |  |
| --- | --- | --- | --- |
| Childcare |  | Leisure activities |  |
| Housework |  | Voluntary work |  |
| Study |  | Inactivity due to ill health |  |
| Other- please give details: |  |  |  |

**B7. Did you travel to the hospital on your own or were you accompanied by another person?** Please tick one box.

Please tick one box.

| By myself |  |
| --- | --- |
| Accompanied by a relative/friend/carer |  |

**B8.** Did anyone help you fill in this questionnaire today?

Yes No

|  |  |  |  |  |  |  |  |  |  |  |
| --- | --- | --- | --- | --- | --- | --- | --- | --- | --- | --- |

**THANK YOU**

**This is the end of the questionnaire. Please take a moment to check that you have answered as many questions as possible.**

**We would like to thank you for your time spent completing the questionnaire. The information you have given us will be extremely useful in helping us carry out our research. It will be treated with the strictest confidence and kept securely.**

***Thank you again for your help***


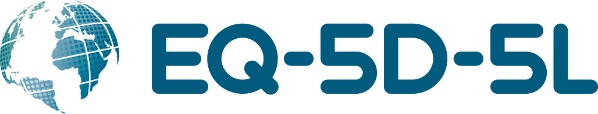


|  |
| --- |
| Health Questionnaire |
|  |
|  |
| English version for the UK |

| Under each heading, please tick the ONE box that best describes your health TODAY. | |
| --- | --- |
| MOBILITY |  |
| I have no problems in walking about | ❑ |
| I have slight problems in walking about | ❑ |
| I have moderate problems in walking about | ❑ |
| I have severe problems in walking about | ❑ |
| I am unable to walk about | ❑ |
| SELF-CARE |  |
| I have no problems washing or dressing myself | ❑ |
| I have slight problems washing or dressing myself | ❑ |
| I have moderate problems washing or dressing myself | ❑ |
| I have severe problems washing or dressing myself | ❑ |
| I am unable to wash or dress myself | ❑ |
| USUAL ACTIVITIES *(e.g. work, study, housework, family or leisure activities)* |  |
| I have no problems doing my usual activities | ❑ |
| I have slight problems doing my usual activities | ❑ |
| I have moderate problems doing my usual activities | ❑ |
| I have severe problems doing my usual activities | ❑ |
| I am unable to do my usual activities | ❑ |
| PAIN / DISCOMFORT |  |
| I have no pain or discomfort | ❑ |
| I have slight pain or discomfort | ❑ |
| I have moderate pain or discomfort | ❑ |
| I have severe pain or discomfort | ❑ |
| I have extreme pain or discomfort | ❑ |
| ANXIETY / DEPRESSION |  |
| I am not anxious or depressed | ❑ |
| I am slightly anxious or depressed | ❑ |
| I am moderately anxious or depressed | ❑ |
| I am severely anxious or depressed | ❑ |
| I am extremely anxious or depressed | ❑ |

The best health you can imagine

| We would like to know how good or bad your health is TODAY. |
| --- |
| This scale is numbered from 0 to 100. |
| 100 means the best health you can imagine. 0 means the worst health you can imagine. |
| Please mark an X on the scale to indicate how your health is TODAY. |
| Now, write the number you marked on the scale in the box below. |

YOUR HEALTH TODAY =

10

0

20

30

40

50

60

80

70

90

100

5

15

25

35

45

55

75

65

85

95

The worst health you can imagine
